# Prohormones for prediction of adverse medical outcome in community-acquired pneumonia and lower respiratory tract infections

**DOI:** 10.1186/cc9055

**Published:** 2010-06-08

**Authors:** Philipp Schuetz, Marcel Wolbers, Mirjam Christ-Crain, Robert Thomann, Claudine Falconnier, Isabelle Widmer, Stefanie Neidert, Thomas Fricker, Claudine Blum, Ursula Schild, Nils G Morgenthaler, Ronald Schoenenberger, Christoph Henzen, Thomas Bregenzer, Claus Hoess, Martin Krause, Heiner C Bucher, Werner Zimmerli, Beat Mueller

**Affiliations:** 1 Department of Internal Medicine, Division of Endocrinology, Diabetes and Clinical Nutrition, University Hospital Basel, Petersgraben 4, 4031 Basel, Switzerland; 2 Basel Institute for Clinical Epidemiology and Biostatistics, University Hospital Basel, Petersgraben 4, 4031 Basel, Switzerland; 3 Oxford University Clinical Research Unit, Wellcome Trust Major Overseas Program, Hospital for Tropical Diseases, Ho Chi Minh City, Vietnam; 4 Department of Internal Medicine, Buergerspital Solothurn, Schöngrünstrasse 38, 4500 Solothurn, Switzerland; 5 Department of Internal Medicine, Kantonsspital Liestal, Rheinstrasse 26, 4410 Liestal, Switzerland; 6 Department of Internal Medicine, Kantonsspital Lucerne, Friedentalstrasse, 6004 Luzern, Switzerland; 7 Department of Internal Medicine, Kantonsspital Muensterlingen, Postfach, 8596 Münsterlingen, Switzerland; 8 Department of Internal Medicine, Kantonsspital Aarau, Tellstrasse, 5000 Aarau, Switzerland; 9 Brahms AG, Neuendorfstraße 25, 16761 Hennigsdorf, Germany

## Abstract

**Introduction:**

Measurement of prohormones representing different pathophysiological pathways could enhance risk stratification in patients with community-acquired pneumonia (CAP) and other lower respiratory tract infections (LRTI).

**Methods:**

We assessed clinical parameters and five biomarkers, the precursor levels of adrenomedullin (ADM), endothelin-1 (ET1), atrial-natriuretic peptide (ANP), anti-diuretic hormone (copeptin), and procalcitonin in patients with LRTI and CAP enrolled in the multicenter ProHOSP study. We compared the prognostic accuracy of these biomarkers with the pneumonia severity index (PSI) and CURB65 (Confusion, Urea, Respiratory rate, Blood pressure, Age 65) score to predict serious complications defined as death, ICU admission and disease-specific complications using receiver operating curves (ROC) and reclassification methods.

**Results:**

During the 30 days of follow-up, 134 serious complications occurred in 925 (14.5%) patients with CAP. Both PSI and CURB65 overestimated the observed mortality (X^2 ^goodness of fit test: *P *= 0.003 and 0.01). ProADM or proET1 alone had stronger discriminatory powers than the PSI or CURB65 score or any of either score components to predict serious complications. Adding proADM alone (or all five biomarkers jointly) to the PSI and CURB65 scores, significantly increased the area under the curve (AUC) for PSI from 0.69 to 0.75, and for CURB65 from 0.66 to 0.73 (*P *< 0.001, for both scores). Reclassification methods also established highly significant improvement (*P *< 0.001) for models with biomarkers if clinical covariates were more flexibly adjusted for. The developed prediction models with biomarkers extrapolated well if evaluated in 434 patients with non-CAP LRTIs.

**Conclusions:**

Five biomarkers from distinct biologic pathways were strong and specific predictors for short-term adverse outcome and improved clinical risk scores in CAP and non-pneumonic LRTI. Intervention studies are warranted to show whether an improved risk prognostication with biomarkers translates into a better clinical management and superior allocation of health care resources.

****Trial Registration**:**

NCT00350987.

## Introduction

The assessment of disease severity and prediction of outcome in lower respiratory tract infections (LRTI) and, in particular, community-acquired pneumonia (CAP), is essential for the appropriate allocation of health care resources and for optimized treatment decisions. These include hospital or intensive care unit admission, the extent of diagnostic work-up, the choice and route of antimicrobial agents and the evaluation for early discharge. In an attempt to optimize and lower unnecessary hospital admission rates, professional organizations have developed prediction rules and propagated guidelines to stratify patients with CAP based on predicted risks for mortality [1-3]. The pneumonia severity index (PSI) is a well validated scoring system in North America based on 19 prognostic parameters [4]. The CURB65 score, a more simplified assessment tool developed by the British Thoracic Society, focuses on only five predictors [5,6]. This score is easier to calculate, but has a lower prognostic accuracy. Both risk scores were validated for the prediction of mortality only. Their ability to predict other important adverse disease outcomes including the need for ICU admission and complications due to the infection has not been established. Patients with PSI risk classes 1, 2 and 3 should be considered as candidates for outpatient treatment, but still a high percentage of subjects in these risk classes may experience unexpected complications indicating the need for improvement of these scores [7].

To improve the accuracy of clinical severity scores, prohormones have been proposed as biomarkers that provide more detailed and complementary information [8-25]. Several biomarkers have been related to disease severity and outcome in LRTI and sepsis, including levels of the cardiac hormone atrial-natriuretic peptide (ANP) [13-17], the stress- and volume-dependent antidiuretic hormone (ADH, vasopressin) [21-25], the endothelium derived hormones endothelin-1 (ET-1) [11,18-20] and adrenomedullin (ADM) [8-12], and procalcitonin (PCT) a specific marker of bacterial infections [26-35].

The simultaneous measurement of a panel of prohormones each reflecting a specific pathophysiological pathway could enhance risk stratification in patients with CAP and other LRTI. We therefore validated the usefulness of five previously reported prohormones for predicting serious complications in patients with CAP and other LRTI enrolled in the multicenter ProHOSP study [31,34].

## Materials and methods

### Study sample

We measured biomarker levels in all patients with LRTIs enrolled in the multicenter ProHOSP study [31]. The design of the ProHOSP study has been reported in detail elsewhere [34]. In brief, from October 2006 to March 2008, a total of 1,359 consecutive patients with presumed LRTIs from six different hospitals located in the northern part of Switzerland were included. Patients were randomly assigned to an intervention group, where guidance of antibiotic therapy was based on PCT cut off ranges or to a standard group where guidance of antibiotic therapy was based on enforced guideline recommendations without knowledge of PCT. The primary end-point in this non-inferiority trial was a combined endpoint of adverse medical outcomes within 30 days following the ED admission. A further predefined secondary objective was the evaluation of different biomarkers to predict serious complications and all causes of mortality as compared to established risk factors and clinical scores.

The study protocol was approved by all local ethical committees, and written informed consent for the collection of blood on admission and during follow-up to measure biomarkers was obtained from all participants.

### Definition of different LRTIs and severity assessment

We used web-based guidelines for a standardized care of patients as defined previously [34]. Thereby, LRTI was defined by the presence of at least one respiratory symptom (cough, sputum production, dyspnea, tachypnea, pleuritic pain) plus at least one finding during auscultation (rales, crepitation), or one sign of infection (core body temperature >38.0°C, shivering, leukocyte count >10 G/l or <4 G/l cells) independent of antibiotic pre-treatment. CAP was defined as a new infiltrate on chest radiograph [1,2,36,37]. Chronic obstructive pulmonary disease (COPD) was defined by post-bronchodilator spirometric criteria according to the Global initiative for chronic Obstructive Lung Disease (GOLD)-guidelines as a FEV1/FVC ratio below 70% [36,38]. Acute bronchitis was defined as LRTI in the absence of an underlying lung disease or focal chest signs and infiltrates on chest x-ray, respectively [37]. The Pneumonia Severity Index (PSI) and the CURB65 scores were calculated in all patients as described on admission to the emergency department [4,6]. Our web-based guidelines provided published criteria for ICU admission based on the 2001 American Thoracic Society (ATS) criteria [1]. In brief, ICU admission should be considered in patients with severe CAP, defined as the presence of either one of two major criteria (need for mechanical ventilation, septic shock), the presence of two of three minor criteria (systolic blood pressure <90 mmHg, multilobar disease, PaO2/FIO2ratio <250) or more than two CURB points. For COPD patients, ICU criteria included severe acidosis or respiratory failure (pO2 <6.7 kPa, pCO2 >9.3 kPa, pH <7.3), no response to initial treatment in the emergency department or worsening mental status (confusion, coma) despite adequate therapy.

### Analysis population, endpoints and covariates

The primary analysis population contains all 925 patients with the final diagnosis of CAP. In a second step, performance of developed models was extrapolated to patients with non-CAP LRTI (that is, acute bronchitis and exacerbation of COPD).

The primary endpoint of this prognostic study was serious complications defined as death from any cause, ICU admission, or disease specific complications defined as local or systemic complications from LRTI including persistence or development of pneumonia (including nosocomial), lung abscess, empyema or acute respiratory distress syndrome within 30 days following inclusion. The secondary endpoint was overall survival within 30 days following study inclusion. Outcomes were assessed during hospital stay at days 3, 5, 7, at hospital discharge, and by structured phone interviews after 30 days by blinded medical students and adjudicated by an independent data-monitoring committee [31,34].

Pre-defined covariates for the prognostic models were the covariates included in the CURB65 score (all covariates except for confusion as continuous variables) and the five prohormones. Prohormone levels and urea were log-transformed prior to all analyses to normalize their distribution. In exploratory analysis we also explored all covariates included in the PSI score.

### Biomarker selection and measurement

We selected five prohormones because of reported associations with death or serious complications, biologic plausibility and availability [8-25]. We measured PCT and proADM as markers of bacterial infection and inflammation; the atrial-natriuretic peptide proANP and proET-1 as markers of cardiac and endothelial function, and the vasopressin precursor copeptin as a marker of stress and fluid balance. ProADM, proET-1, proANP and copeptin were batch-measured in plasma with new sandwich immunoassay as described elsewhere [8,25,39-41]. The assays have analytical detection limits of 0.08 nmol/L, 0.4 pmol/L, 4.3 pmol/L and 0.4 pmol/L, respectively. PCT was measured with a high sensitive time-resolved amplified cryptate emission (TRACE) technology assay (PCT Kryptor^®^, B.R.A.H.M.S. AG, Hennigsdorf, Germany). The assay has a detection limit of 0.02 μg/L and functional assay sensitivity of 0.06 μg/L.

### Statistical analysis

#### Development and assessment of prognostic models

To assess the univariate predictive potential of the five biomarkers and all covariates included in the PSI and CURB65 scores on the endpoints we first calculated the areas under the ROC curve (AUCs) for each covariate separately. The univariate association between the two most predictive biomarkers, proADM and proET1, respectively, and the risk of a serious complication and death, respectively, was also estimated using a generalized additive model. In addition, we assessed the calibration of the PSI and CURB65 scores using X^2 ^goodness of fit tests. Expected risks for these scores were based on the risks reported in the original PSI and CURB65 publications [4,6]. In both cases, we used observed risks from all patients (derivation and validation cohorts) from those studies.

Second, we assessed the significance and improvement in AUCs if biomarkers were included into a logistic model in addition to either the CURB65 or the PSI risk score. Third, we fitted the three predefined multivariable logistic regression models for the two separate endpoints, that is, serious complications and death. The models contained the CURB65 covariates alone, jointly with proADM, and jointly with all remaining biomarkers. Analyses for both endpoints address the limitation that the CURB65 and PSI scores were originally designed to assess mortality risks as the main outcome. In order to avoid over-fitting in view of the limited number of patients reaching the endpoints we restricted this analysis to covariates from the CURB65 score. Further, we chose to look at proADM separately because it had the best track record based on earlier publications [8-12]. In addition, we assessed how well the multivariable models, which were developed for CAP patients only, extrapolate to patients without CAP.

The performance of the prognostic models was assessed by ROC curves, the AUC and the mean Brier score. The Brier score for the i^th ^individual is the squared difference between his predicted probability of an event and the outcome (0 = no event, 1 = event). The mean Brier score is the average Brier score amongst all patients. For an individual, the Brier score can range from 0 (concordant prediction and outcome) and 1 (discordant prediction and outcome); a prediction of 50% has a score of 0.25 both when the outcome is 0 or 1 [42].

The development and assessment of prognostic models based on the same dataset may lead to over-fitting and thus over-optimistic conclusions. To avoid this bias we used for all performance measures optimism-corrected bootstrap validation with 1,000 bootstrap replications [42,43]. Because the study recruited patients from six different hospitals, we additionally performed six-fold cross-validation and fitted the model based on data from five hospitals, to evaluate performance on patients from the remaining hospital. The average performance measure over all six left-out hospitals provides a conservative estimate of average performance on a similar hospital to those included in the study. ROC curves were optimism-corrected or cross-validated by vertical averaging, that is, by averaging over true positive rates at fixed false positive rates. For comparing the model with all CURB65 covariates vs. the model with CURB65 covariates and all five biomarkers, we also assessed reclassification by reclassification tables (for risk cut-offs at 5%, 10%, and 20%), net reclassification improvement and integrated discrimination improvement [44]. These measures were either based on predictions from a model fit on the full dataset or, as a sensitivity analysis, on out-of-sample predictions from leave-one-hospital-out cross-validation as described above. In both cases, we used the average predicted risks over all imputed datasets (see below).

Finally, we assessed the additional prognostic value of prohormones on Days 3, 5, and 7 of follow-up, respectively, by modeling the time to the first serious complication as depending on the initial prohormone value as well as the time-updated biomarker value using the Cox proportional hazards regression models with time-dependent covariates.

#### Treatment of missing values

We used multiple imputations by chained equations to deal with missing covariate and biomarker values. The imputation dataset consisted of all 1,359 ProHOSP patients (that is, including CAP and non-CAP LRTI) and the following variables: All covariates included in the derivation of the PSI or CURB65 risk scores, biomarkers values on Days 0, 3, 5, and 7, randomization arm, final diagnosis, total antibiotics exposure, length of hospital stay as well as death, ICU admission, complication, or disease recurrence within 30 days of randomization. Outcomes were also included in the imputation to avoid bias. All reported results were aggregated over five imputed datasets except for the time-dependent Cox regression, which was based on the first imputed dataset only.

#### Statistical software

All analyses were performed with R 2.5.1 (R Foundation for Statistical Computing, Vienna, Austria). We used the contributed R packages mice for imputation of missing values, and ROCR for ROC analysis [45-47].

## Results

### Patient population

A total of 1,359 persons with the presumed diagnosis of LRTI were included. A majority of patients (92.5%) were admitted to the hospital with a median length of stay of eight (interquartile range (IQR) 4 to 12) days. CAP was diagnosed in 925 patients, which is the primary population studied in this analysis. Exacerbation of COPD was diagnosed in 228, acute bronchitis in 151, and 55 patients had another final diagnosis than LRTI. During the 30 days of follow-up, 170 patients (12.5%) with LRTI had at least one serious complication including death in 67 patients (4.9%), need for ICU admission in 103 patients (7.6%) and development of empyema in 31 patients (2.3%). Most serious complications occurred in the 925 patients with CAP (n = 134, 14.5%). In CAP patients, death occurred in 50 patients (5.4%), need for ICU admission in 83 patients (8.9%) and disease-specific complications, which consisted of empyema only, in 31 patients (3.4%). Of note, some patients experienced more than one serious complication. The number of patients with CAP in the six participating centers ranged between 122 and 210 with between 19 and 28 serious complications per center. Baseline characteristics and median levels of the biomarkers in primary analysis population (CAP patients) are presented in Table 1. Biomarkers were all positively inter-correlated with rank correlations ranging from 0.23 (between PCT and ProANP) to 0.87 (between proET1 and proADM).

**Table 1:** Characteristics of CAP patients at admission (n = 925)

Characteristics	All CAP patients (n = 925)	Serious complications (n = 134)	No serious complications (n = 791)	*P*	AUC
**Demographic characteristics**					
-Age (years)*	72 (59 to 82)	74 (62 to 82)	72 (58 to 82)	0.33	0.53
- Sex (male) - no. (%)	544 (58.8)	87 (64.9)	457 (57.8)	0.12	

**Coexisting illnesses **- no. (%)					
-Coronary heart disease	183 (19.8)	38(28.4)	145 (18.3)	0.007	-
-Renal dysfunction	206 (22.3)	58 (43.3)	148 (18.7)	<0.001	-
-COPD	282 (30.5)	58 (43.3)	224(28.3)	0.001	-

**Clinical findings**					
-Confusion - no. (%)	87 (9.4)	19 (14.2)	68 (8.6)	0.04	-
-Respiratory rate (breaths/minute)*	20 (16 to 25)	24 (18 to 30)	20 (16 to 25)	<0.001	0.63
-Systolic blood pressure (mmHg)*	132 (119 to 148)	120 (105 to 140)	134 (120 to 150)	<0.001	0.62
-Heart rate (beats/minute)*	95 (82 to 108)	99 (81 to 114)	94/102 to 106)	0.02	0.56
-Body temperature (C°)*	38.1 (37.2 to 38.9)	38.0 (37.1 to 38.7)	38.1 (37.3 to 38.9)	0.19	0.53

**Biomarkers**					
-Procalcitonin (μg/l)*	0.71 (0.44 to 1.53)	1.12 (0.66 to 2.39)	0.66 (0.43 to 1.41)	<0.001	0.66
-ProADM (nmol/l)*	1.1 (0.9 to 1.3)	1.4 (1.1 to 1.8)	1.1 (0.9 to 1.3)	<0.001	0.72
-ProANP (pmol/l)*	9.1 (7.1 to 12.1)	11.2 (8.2 to 14.4)	8.7 (6.7 to 11.7)	<0.001	0.65
-ProET1 (pmol/l)*	7.8 (6.7 to 9.3)	9.6 (7.6 to 11.3)	7.6 (6.6 to 8.9)	<0.001	0.72
-Copeptin (pmol/l)*	4.0 (3.0 to 5.5)	5.4 (4.0 to 8.2)	3.8 (2.9 to 5.2)	<0.001	0.70

**Risk assessment at admission**					
-PSI points*	94 (67 to 116)	116 (95 to 141)	91/67 to 116)	<0.001	0.69
-PSI class*	4 (2 to 4)	4 (4 to 5)	4 (2 to 4)	<0.001	0.67
-CURB-65 points*	2 (1 to 2)	2 (1 to 3)	2 (1 to 2)	<0.001	0.66

Baseline characteristics based on first imputed dataset. *P*-values according to Wilcoxon rank sum test or chi-square test, respectively. AUCs correspond to averaged results over all imputed datasets and were calculated for continuous characteristics only.

CAP, community-acquired pneumonia; PSI, pneumonia severity index; CURB65, confusion, uremia, respiratory rate, blood pressure, age 65 years or greater; AUC, area under the ROC curve; *expressed as median (interquartile range, IQR).

All biomarkers on admission were available in 94.8% of patients. The most frequently missing covariate contained in the CURB65 score was urea which was missing in 19.1% of patients, primarily because it was only rarely measured in one participating hospital. The number of patients with a complete assessment of CURB65 covariates and biomarkers at baseline was 539 (58%). In patients who were alive and remained in hospital until the respective follow-up day, all biomarker values on Days 3, 5, and 7 of follow-up were available in 91.1%, 87.6% and 86.1% of patients, respectively.

### Calibration of PSI score and CURB65 score

Both PSI and CURB65 significantly overestimated the mortality risk in CAP patients (*P = 0.003 *and *0.01 *for X^2 ^goodness of fit test). This overestimation occurred in almost all risk categories (Table 2) and also in all hospitals. Only one death was observed in 423 patients with PSI Classes 1 to 3. In contrast, patients in PSI Class 1 had already a 4.8% incidence of serious complications.

**Table 2:** Predicted and observed number of events according to PSI and CURB65 risk category in CAP patients (n = 925)

PSI class	1	2	3	4	5
Predicted death risk (%)*	0.18%	0.63%	2.74%	8.31%	29.62%

Observed data					
- n	104	139	180	351	151
- Number of deaths	0 (0.0%)	0 (0.0%)	1 (0.6%)	23 (6.6%)	26 (17.2%)
- Number of ICU or death	4 (3.8%)	7 (5.0%)	8 (4.4%)	55 (15.7%)	44 (29.1%)
- Number of serious complications	5(4.8%)	12 (8.6%)	13 (7.2%)	60 (17.1%)	44 (29.1%)

**CURB65 score**	0	1	2	3	4 or 5

Predicted death risk (%)*	0.58%	1.66%	9.02%	16.11%	35.10%

Observed data					
- n	194	233	296	167	35
- Number of deaths	0 (0.0%)	4 (1.7%)	25 (8.4%)	10 (6.0%)	11 (31.4%)
- Number of ICU or death	6 (3.1%)	19 (8.2%)	44 (14.9%)	31 (18.6%)	18 (51.4%)
- Number of serious complications	7 (3.6%)	30 (12.9%)	46(15.6%)	33(19.8%)	18(51.4%)

* Based on risks reported in the original PSI and CURB65 publications (derivation and validation cohorts) [4,6].

### Univariate discriminatory power of biomarkers

Discriminatory power of biomarkers for predicting serious complications in CAP patients as assessed by the area under the ROC curve (AUC) ranged from 0.66 for proANP to 0.72 for proADM and proET1 (Table 1). Of note, the best biomarkers had higher AUCs than the CURB65 (AUC = 0.66) or the PSI score (AUC = 0.69) as well as all individual covariates included in these scores.

Discriminatory power of biomarkers for predicting death ranged between 0.60 for PCT to 0.76 for proADM and 0.79 for proANP. CURB65 and PSI score had AUCs of 0.74 and 0.84, respectively. Again, the best biomarker had a higher AUC than all covariates included in the CURB65 or PSI scores (data not shown).

**Figure 1:**
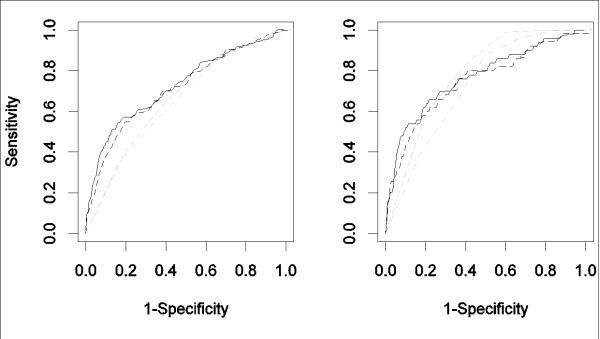
**Univariate association of the biomarkers with serious complications (left panel) and death (right panel)**. ProADM (black, solid line), proET1 (black, dashed line), PSI class (grey, dashed line) and CURB65 score (grey, dash-dotted line).

**Figure 2:**
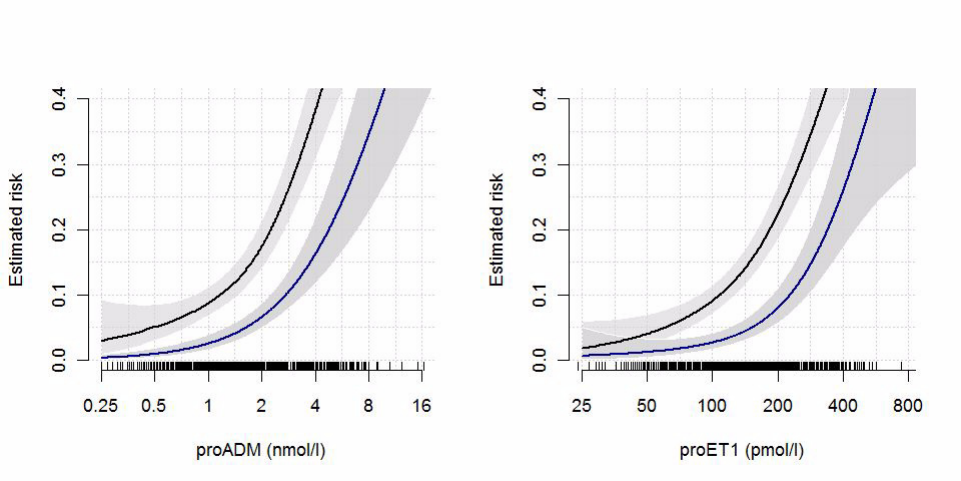
**Estimated association of proADM and proET1 levels with risk of serious complications (upper black line) and death (lower blue line)**. Estimates are based on generalized additive models and shaded gray regions correspond to (point-wise) 95% confidence intervals. The rugs at the bottom of the plots display the distribution of the biomarker.

Corresponding ROC curves are displayed in Figure 1 (all biomarkers, PSI and CURB65). Figure 2 displays the estimated association of the prohormones proADM and proET1 with the risk of serious complications and death, respectively.

### Discriminatory power of biomarkers adjusted for risk scores

A combination of proADM in a logistic regression model with either the CURB65 or the PSI risk score for the prediction of serious complications yielded significant effects for proADM (both *P *< 0.001); the odds ratio by one standard deviation increase of log-proADM was 2.11 (95% CI 1.69 to 2.64) and 1.98 (95% 1.59 to 2.47) for the two models, respectively. Likewise, the AUC (as assessed by six-fold cross-validation) increased from 0.66 to 0.73 and from 0.69 to 0.75, respectively. Adding all biomarkers instead of proADM alone did not lead to a further improvement of the models (*P *= 0.19 and 0.15, respectively). Results were similar for a complete-case analysis which did not impute any missing data (*P *< 0.001 for proADM combined with CURB65 and *P *= 0.004 for proADM combined with the PSI score).

For predicting mortality in CAP patients, the addition of proADM to CURB65 or PSI, respectively, was again significant (both *P *< 0.001) with odds ratios of 2.08 (95% CI 1.52 to 2.85) by one standard deviation increase of log-proADM and 1.76 (95% CI 1.27 to 2.42), respectively. The AUC increased from 0.74 to 0.80 and from 0.84 to 0.86, respectively. Adding all biomarkers instead of proADM alone lead to a further improvement of the model for CURB65 (*P *= 0.03) but not for the PSI (*P *= 0.38).

### Multivariable statistical models

The multivariable logistic model for the primary and secondary endpoint in CAP patients with all CURB65 covariates and proADM is displayed in Table 3. Note that for the primary endpoint older patients are less likely to experience serious complications after adjustment for other covariates.

**Table 3:** Logistic model for the prediction of serious complications or death using proADM and all CURB covariates

	Serious complications			Death		
		
	OR	95% CI	*P*	OR	95% CI	*P*
Intercept	0.08	(0.06, 0.11)	<0.001	0.02	(0.01, 0.03)	<0.001
Confusion - yes	2.05	(1.07, 3.91)	0.03	2.30	(1.01, 5.22)	0.047
Urea (by two-fold increase)	1.59	(1.05, 2.41)	0.03	1.51	(0.85, 2.70)	0.16
Respiratory rate (by +10 breaths/minute)	1.38	(1.10, 1.74)	0.01	1.23	(0.88,1.72)	0.23
Systolic blood pressure (by +10 mmHg)	0.90	(0.82, 0.98)	0.02	0.90	(0.79, 1.03)	0.11
Age (by +10 years)	0.82	(0.71, 0.95)	0.01	1.62	(1.18, 2.23)	0.003
ProADM* (by 2-fold increase)	1.92	(1.44, 2.57)	<0.001	1.84	(1.18, 2.87)	0.01

OR, Odds ratio, CI, Confidence interval.

Intercept corresponds to a person without confusion, urea of 7 mmol/l, respiratory rate of 20 breaths/minute, systolic blood pressure of 130 mmHg, age 70 years and ProADM of 1 nmol/l.

* OR (95% CI, *P*-value) for proADM in the complete case analysis without imputation of missing data are 1.61 (1.13 to 2.31; *P *= 0.01) for the prediction of serious complications and 1.65 (0.97 to 2.79; *P *= 0.06) for the prediction of death.

**Figure 3:**
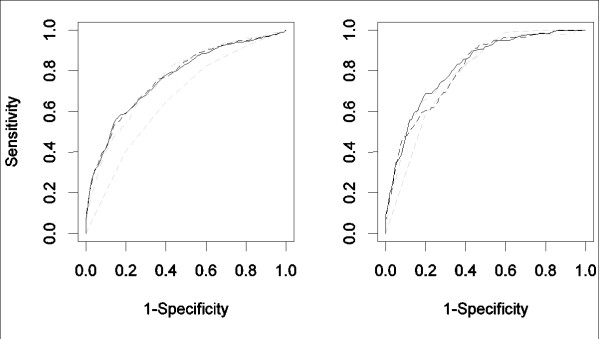
**ROC curves of multivariable models for the prediction of serious complications (left panel) and death (right panel) during 30 days of follow-up**. Models are based on CURB65 covariates alone (grey, dash-dotted lines), or jointly with proADM (black, solid lines) or all five biomarkers (black, dashed lines), respectively, ROC curve estimated by six-fold cross-validation (leave-one-hospital out). The predictive accuracy of the PSI class (gray, dashed lines) is added as a comparison.

ROC curves for all pre-defined multivariable models for the prediction of serious complications and mortality in CAP patients and corresponding performance measures are displayed in Table 4 and Figure 3. All multivariable models improved the prediction of serious complications as compared to the PSI score and CURB covariates. However, the differences between the three multivariable models according to the AUC and the Brier score appeared to be small. Cross-validated AUC's for the model based on CURB65 covariates and proADM ranged between 0.72 to 0.81 for the respective hospital that was left-out from the model fitting. The cross-validated AUC of 0.73 and Brier score of 0.14 for the center which had urea missing for almost all patients tended to be poorer than for other hospitals.

**Table 4:** Performance of multivariable models for the prediction of death, ICU or complication in CAP patients (n = 925)

Endpoint	Model	Bootstrap-corrected accuracy measure	Leave-one-hospital-out cross-validation Accuracy calculated on left out hospital	
			
			Mean	Range
Serious complication*	CURB covariates			
	-AUC	0.75	0.75	0.67 to 0.83
	-Brier score	0.11	0.11	0.09 to 0.15
	
	CURB covariates + proADM			
	-AUC	0.76	0.76	0.72 to 0.81
	-Brier score	0.10	0.11	0.09 to 0.14
	
	CURB covariates + all biomarkers			
	-AUC	0.76	0.76	0.71 to 0.81
	-Brier score	0.11	0.11	0.09 to 0.14

**Death**	CURB covariates			
	-AUC	0.80	0.81	0.72 to 0.87
	-Brier score	0.05	0.05	0.03 to 0.07
	
	CURB covariates + proADM			
	-AUC	0.81	0.82	0.71 to 0.87
	-Brier score	0.05	0.05	0.03 to 0.07
	
	CURB covariates + all biomarkers			
	-AUC	0.80	0.81	0.72 to 0.88
	-Brier score	0.05	0.05	0.03 to 0.07

A reclassification [44] table of the model with CURB65 covariates only vs. the model with CURB65 covariates and biomarkers is shown in Table 5. Reclassification methods showed significant benefit from adding biomarkers to clinical covariates. Specifically, net reclassification improvement and integrated discrimination improvement were 0.17 (*P *< 0.001) and 0.04 (*P *< 0.001), respectively, if based on predictions derived on the full dataset, and 0.13 (P = 0.01) and 0.04 (*P *< 0.001), if based on out-of-sample predictions from leave-one-hospital out cross-validation.

**Table 5:** Reclassification table for serious complications in clinical covariates only model compared to clinical covariates plus all biomarkers model

	Model with clinical covariates and all biomarkers					
**Model with clinical covariates only**	**Risk categories**	**≤5%**	**>5 to 10%**	**>10 to 20%**	**>20%**	**Row total**
	
	**≤5%**					
	**Number**	116	22	3	0	**141**
	**Actual risk**	0.04	0.00	0.33	-	**0.04**
	
	>5 to 10%					
	**Number**	96	160	46	2	**304**
	**Actual risk**	0.03	0.04	0.09	0.5	**0.05**
	
	**>10 to 20%**					
	**Number**	3	96	154	34	**287**
	**Actual risk**	0.33	0.08	0.18	0.32	**0.16**
	
	**>20%**					
	**Number**	0	1	32	160	**193**
	**Actual risk**	-	0.00	0.09	0.39	**0.34**

**Column total**						
	**Number**	**215**	**279**	**235**	**196**	
	**Actual risk**	**0.04**	**0.05**	**0.15**	**0.38**	

### Prognostic value of biomarker values measured during follow-up

**Figure 4:**
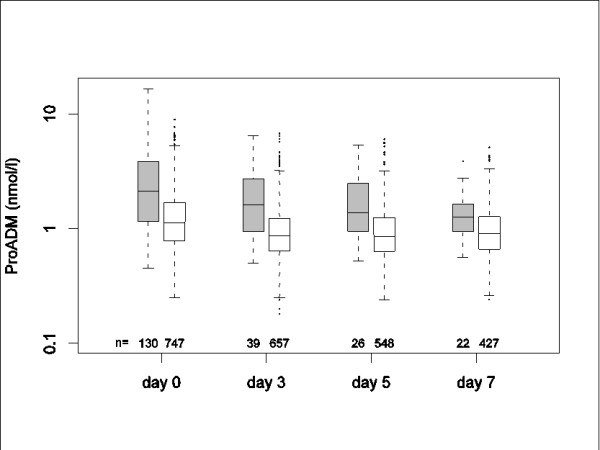
**Boxplots of measured ProADM levels on admission (Day 0) and during follow-up (Days 3, 5, and 7) in patients with serious complications (boxplots with grey filling) and those without (boxplots with white filling)**. n refers to the number of available ProADM measurements at the respective time point (for example, on admission, ProADM was available in 130/134 patients with serious complications). Only ProADM values before the first serious complication were included in patients with complications.

Boxplots of measured ProADM levels on admission and during follow-up in patients with and without serious complications are displayed in Figure 4. Sixty-eight percent (91/134) of first serious complications, particularly ICU admission, occurred within two days of randomization, that is, prior to the first scheduled follow-up visit on day 3.

The hazards for the time to the first serious complication depending on the initial ProADM level or the time-updated ProADM level, were increased by 2.23 (95% CI 1.91 to 2.61) and 2.44 (95% CI 2.08 to 2.85) per two-fold increase in ProADM. When both the initial and the time-updated value of ProADM were included in the model, initial ProADM did not remain a significant predictor (*P *= 0.49), whereas the time-updated value remained significant (*P *< 0.001) suggesting that the latter is a better predictor for future serious complications. The same was found when the Cox regression was additionally adjusted for the CURB65 covariates.

Findings for other biomarkers were consistent. For all biomarkers, the time-updated value was a stronger predictor than the initial value though for PCT and copeptin also the initial value of the marker remained significant in the model with both the initial and the time-updated marker (*P *= 0.046 and *P *= 0.03, respectively).

### Performance of multivariable statistical models in LRTI patients without CAP

The multivariable models for predicting serious complications developed in CAP patients extrapolated well if evaluated in 434 patients with presumed other LRTI in the ProHOSP trial. The AUCs for these patients and the model with all CURB65 covariates and proADM, or with all biomarkers, respectively, were both 0.80 and thus better than on the original population. There was also no indication of serious miscalibration of these models: A total of 36 serious complications were observed in non-CAP patients compared to predicted numbers of complications of 41.2 and 40.2 patients according to the two models, respectively (*P *= 0.39 and *P *= 0.48 for X^2 ^goodness of fit test). The model with only clinical covariates extrapolated worse with an AUC of 0.75 in non-CAP patients and some evidence of miscalibration with 49.7 predicted events (*P *= 0.04).

## Discussion

In this large community-based sample of patients with CAP and other LRTI from a multicenter study [34], five prohormones from distinct biologic pathways were specific predictors for short term serious complications with moderate improvement of clinical risk scores. Thereby, this study validates a series of previous smaller trials demonstrating a clinical utility of prohormones for an optimized risk prediction in LRTI [8-25].

Meaningful statistical assessment of the potential clinical utility of a biomarker is challenging. In addition to classical performance measures like two group comparisons and ROC curves, more clinically meaningful statistical approaches have been put forward [44,48]. We performed several different statistical analyses to investigate the added value of biomarkers to clinical scores; more specifically, we assessed the addition of prohormones to PSI and CURB65 scores *per se *and to a multivariate regression model based on CURB65 covariates. We measured the prognostic performance of these models by several different quantities (AUC, Brier score and reclassification methods). Thereby, some prohormones, namely proADM, improved both clinical risk scores and were superior per se for serious complications prediction. The incorporation of a combination of biomarkers reflecting systemic inflammation, endothelial dysfunction, stress and cardiac function to the clinical risk scores improved their prognostic accuracy for prediction of short term complication rate and to a lesser extent mortality. When comparing the biomarkers to models based on raw clinical predictors included in the CURB65 score, the improvement was less extensive as shown by a relatively small increase in the AUC, but reclassification methods still established highly significant improvements of the model due to addition of the prohormones. Thus, as demonstrated previously for biomarkers in cardiovascular disease [44], prohormones significantly improve classification of patients into pre-defined risk groups.

The combination of clinical predictors and prognostic biomarkers has been suggested as a promising approach to optimize the prognostic certainty and thus the management of LRTI patients [49]. The information on the disease driven host-response mirrored in the circulating level of a biomarker may provide insights into the pathophysiology and prognosis of a disease process. As a quantifiable tool it facilitates risk stratification and monitoring of therapy as a surrogate outcome measure. In the future, a panel of biomarkers might help in delineating distinct populations of patients with discrete pathologies - a prerequisite to enable the targeted application of specific biologically rational therapies. In this trial, we validate the prognostic performance of five promising, rapidly measurable prohormones [8-25]. ADM is one of the most potent vasodilating agents with immune modulating, metabolic and bactericidal properties [40,50]. Atrial-natriuretic peptide, a member of the family of natriuretic peptides regulates a variety of physiological parameters [51]. In septic states, ANP levels may mirror both, the inflammatory cytokine response correlated with the severity of infection, as well as the presence of disease-relevant comorbidities, namely heart failure and renal dysfunction [41,52]. Copeptin, stoichiometrically cleaved from the vasopressin precursor, has hemodynamic and osmoregulatory effects, and mirrors the individual stress response [53]. Endothelin-1 is an important vasoconstrictor and correlates with disease severity and short term outcome [11,18-20]. Unfortunately, these mature hormones are difficult to measure with high reliability because they are not stable at room temperature and have a rapid clearance from the circulation limiting their use in clinical routine. For this reason new sandwich immunoassays have been recently introduced that measure the more stable precursor fragments (proANP, Copeptin (proADH), proET-1 and proADM) [8,25,39-41]. Unlike the mature peptides, these precursors can be detected for hours in the circulation. Because of the stoichiometric generation, these prohormones correlate with the release of the active peptides, a condition similar to that of insulin and C-peptide. Thus, these precursor peptides can be used to indirectly measure the release of the mature hormone under physiological and pathological conditions.

We focused our analysis on initial risk assessment and initial prohormone levels, but also explored the utility of repeated biomarker measurements. We used Cox proportional hazards regression models with time-dependent covariates (in addition to the baseline biomarker) and found that this model significantly improves upon the model with baseline covariates only. Moreover, we found that the baseline value of the biomarker is no longer significant after adjustment for the current biomarker value suggesting that the absolute value of the current biomarker value contains most information regarding future prognosis and the baseline value (as well as the change in the biomarker from baseline to follow-up) are less relevant. Further research is needed to derive clinical decision rules based on time-updated biomarker values.

The development of sepsis from a localized infection is a dynamic continuum and in the majority a sequelae of CAP [54]. The severity of a disease determines the consumption of costly and limited health-care resources. An early and adequate diagnosis and risk assessment is, thus, pivotal for optimized risk-adapted care of patients with severe infections. Scoring systems, such as the PSI, are well validated prognostic tools to determine mortality risks and rely mostly on age as the main driver of mortality [4]. However, calculation of the PSI in daily practice is time consuming which limits its dissemination and implementation in routine care [55]. In addition, the PSI is not a validated predictor for the clinically relevant rate of serious complications. Other clinical prediction rules have focused to predict eligibility for ICU admission. Multiple ICU prediction rules have been proposed including the Infectious Disease Society of America/American Thoracic Society (IDSA/ATS) criteria, the SMART-COP and scores based on the PIRO (Predisposition, insult/infection, response, and organ dysfunction) concept [56-60].

We focused our analysis on a combined endpoint of serious complications, which included mortality, ICU admission and disease-specific complications. The strength of this approach is the clinical relevance for initial site-of-care decisions as patients experiencing one of these serious complications should arguably not be managed in the outpatient setting. However, heterogeneity of this combined endpoint makes prognostication more challenging as shown by the lower AUCs in ROC curves in this study when compared to mortality prediction alone. While age and comorbidities are major drivers of mortality, extent and severity of infection and organ failure may be the most important predictors for ICU admission. In this regard, combination of clinical parameters and biomarkers seems a promising approach.

As a limitation of this study, our findings may not unconditionally be applied to a general LRTI population because of selection bias in regard to exclusion criteria of the underlying randomized controlled trial. Since the PCT-guided group in the ProHOSP trial was non-inferior to the guidelines group with respect to the risk of adverse outcomes, treatment assignment was not considered any further in this analysis. Switzerland has previously been shown to have very low rates of ICU-acquired nosocomial infections and related mortality; thus country-specific differences may limit generalizability and external validation is warranted [61]. Further, covariates for risk score determination (but not biomarker values) were relatively frequently missing in our dataset. We used multiple imputations to deal with missing variables, but this methodology may not be correct on an individual patient basis and may explain some of the PSI and CURB65 miscalibration observed within this study. Although we provided web-based guidelines based on ATS criteria for ICU admission of patients, the final decision for ICU admission was left to the treating physician team. Other clinical risk scores have been suggested for prediction of ICU admission [56-60]. However, as not all covariates were prospectively collected we did not compare biomarkers with these scores.

Previous studies have demonstrated the clinical and scientific impact of the biomarker PCT on the antibiotic management of LRTI [26-32] but up to now, no study has investigated the clinical utility of a prognostic biomarker on the management of patients with LRTI. Because of its high prevalence and associated large need of health care resources, accurate prognostication and improved site-of-care decisions have high relevance for public health, both for primary and hospital care. The ultimate clinical utility of a biomarker is defined by the degree it improves clinical decision making and adds timely information beyond that of readily available information from clinical examination. Observational studies alone cannot provide such information, but may help to provide a rationale for future intervention studies. These are now warranted to show whether biomarker measurement improves risk prognostication and thus the clinical management of patients with LRTI.

## Conclusions

Our data suggest that the addition of a panel of prohormones of distinct biological pathways, particularly proADM, to established CAP risk scores improves the risk stratification for serious complications and mortality in CAP patients. If these results are validated, the incorporation of one or several biomarkers in clinical practice for the prediction of adverse prognosis could help to optimize admission decisions in patients with LRTI and CAP upon hospital admission and in the emergency department.

## Key messages

• The simultaneous measurement of different prohormones each reflecting distinct pathophysiological pathways could enhance risk stratification in patients with CAP.

• The precursor levels of adrenomedullin (ADM), endothelin-1 (ET1), atrial-natriuretic peptide (ANP), anti-diuretic hormone (copeptin), and procalcitonin (PCT) on admission and during follow up, showed all high prognostic accuracies for mortality and serious complications.

• For the prediction of serious complications, ProADM and proET1 alone had stronger discriminatory power than the PSI or CURB65 score or any components of these risk scores. The inclusion of proADM alone (or all five biomarkers jointly) in addition to the PSI or CURB65 scores, respectively, significantly improved the AUC for prediction of serious complications.

• Reclassification methods also established highly significant improvement for models with biomarkers compared to models based on clinical covariates only.

• Future intervention studies are warranted to show whether an improved risk prognostication with biomarkers translates into a better clinical management and superior allocation of health care resources.

## Abbreviations

ADH: antidiuretic hormone; ADM: adrenomedullin; ANP: atrial-natriuretic peptide; ATS: American Thoracic Society; AUC: area under the ROC curve; CAP: community-acquired pneumonia; COPD: chronic obstructive pulmonary disease; CURB65: Confusion: Urea: Respiratory rate: Blood pressure: Age 65; ET-1: Endothelin-1; ICU: intensive care unit; IDSA: Infectious Disease Society of America; IQR: interquartile range; LRTI: lower respiratory tract infection; PCT: procalcitonin; PIRO: predisposition: insult/infection: response: and organ dysfunction; PSI: pneumonia severity Index; ROC: receiver operating characteristics; TRACE: time-resolved amplified cryptate emission.

## Competing interests

No commercial sponsor had any involvement in design and conduct of this study, namely, the collection, management, analysis, and interpretation of the data; and preparation, decision to submit, review, or approval of the manuscript.

NGM is employed by BRAHMS AG, part of Thermo Fischer Scientific, a company which develops *in vitro *diagnostica, including some of the biomarkers mentioned in this study. PS, MCC and BM received support from BRAHMS to attend meetings and fulfilled speaking engagements. BM has served as a consultant and received research support from BRAHMS.

## Authors' contributions

PS, BM and MCC had the idea and initiated the study. MCC, WZ, HCB, MW, PS, US, and BM designed the study and wrote the protocol. PS, US, KR, RT, CF, IW, SN, CB, TF, RS, CH, TB, CH, MK, WZ and BM managed the trial and collected data. NGM was responsible for the biomarker measurements in a blinded way. The statistical analyses were performed by MW and PS. PS and MW take full responsibility for the reported results. PS, MW and BM drafted the manuscript. All authors amended and commented on the manuscript and approved the final version.
